# Fracture through Pre-Existing Tarsal Coalition: A Narrative Review

**DOI:** 10.3390/children10010072

**Published:** 2022-12-29

**Authors:** Albert T. Anastasio, Emily M. Peairs, Caitlin Grant, Billy I. Kim, Anthony Duruewuru, Samuel B. Adams

**Affiliations:** 1 Department of Orthopaedic Surgery, Duke University Medical Center, Durham, NC 27705, USA; 2 Duke University School of Medicine, Durham, NC 27705, USA; 3 Baylor College of Medicine, Houston, TX 77030, USA

**Keywords:** tarsal coalition, calcaneonavicular coalitions, talocalcaneal coalition, ankle, foot, biomechanics, stress fracture

## Abstract

Tarsal coalitions are abnormal fibrous or bony connections between the tarsal bones of the foot. While not always symptomatic, coalitions can cause pain, alterations in forefoot and hindfoot morphology, and alterations in foot and ankle biomechanics. Previous research has described the association of tarsal coalitions with fractures of the lower extremity. Multiple reports of acute fracture in the presence of tarsal coalition have been presented, as have reports of stress fractures of the foot and ankle with concomitant coalition, insidious in onset and thought to be related to aberrancies in foot and ankle biomechanics. The purpose of this review is to discuss the biomechanics seen in tarsal coalitions and to describe reports of fracture occurring concomitantly with tarsal coalitions. We will discuss diagnostic options and treatment approaches in the setting of fracture with preexisting tarsal coalition.

## 1. Introduction

**Figure 1:**
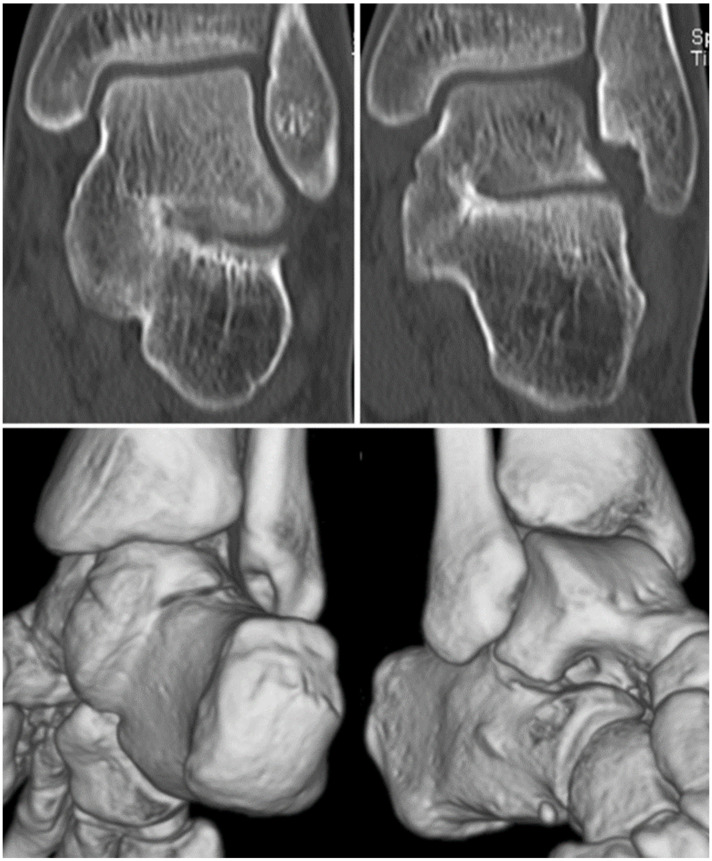
Talocalcaneal coalition, as viewed from two coronal CT sections (upper images) and three-dimensional (3D) reconstruction (lower image). Abnormal bony coalition is noted at the medial aspect of the posterior facet of the subtalar joint [10] (Borrowed using Creative Commons licensing).

**Figure 2:**
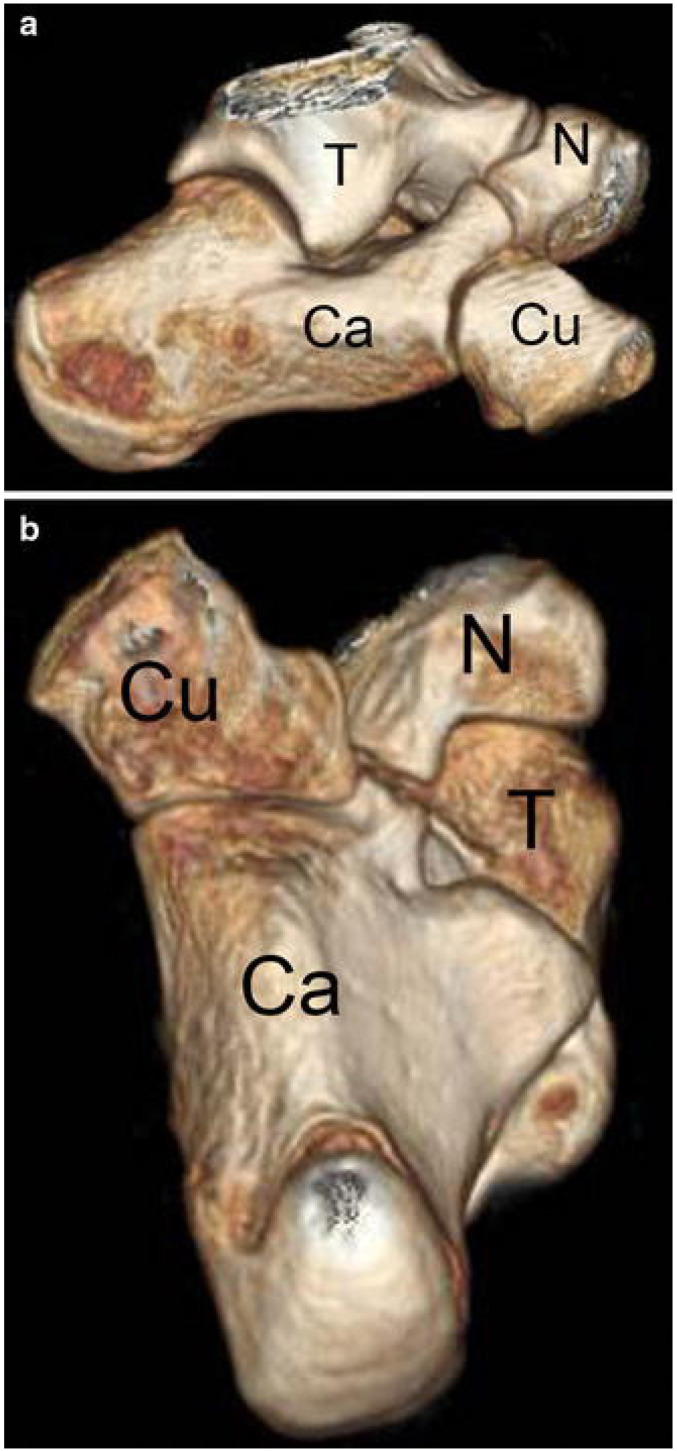
Cropped 3D reconstruction demonstrating sagittal (**a**) and axial (**b**) views of a calcaneonavicular coalition. (calcaneus (Ca), navicular (N), talus (T) and cuboid (Cu)) [11] (borrowed using Creative Commons licensing).

A tarsal coalition is an abnormal connection of the tarsal bones of the foot [1,2]. This bridging can be fibrous, cartilaginous, or bony in origin, and most often involves the talocalcaneal (Figure 1) or calcaneonavicular joints (Figure 2) [1,3]. Other more rare tarsal coalitions include talonavicular, cubonavicular, naviculocunieform, and calcaneocuboid [3,4]. Congenital coalitions occur in an autosomal dominant fashion from a failure of early mesenchymal differentiation [2,4]. Recent research has identified a proline to arginine mutation in the fibroblast growth factor receptor 3 (*FGFR3*) gene in association with these malformations [5]. While tarsal coalitions can be associated with other congenital disorders such as Apert syndrome or Nievergelt-Pearlman syndrome, it is more common for these abnormalities to be isolated occurrences [6,7,8,9]. Acquired tarsal coalitions can be caused by degenerative joint disease, inflammatory arthritis, infectious sequelae, or clubfoot deformities [1,4,6].

Commonly, these coalitions present in childhood and adolescence with vague hindfoot pain or recurrent sprains and other minor injuries [1,6]. Calcaneonavicular coalitions typically present earlier, from 8 to 12 years of age, while talocalcaneal present in patients 12–16 years of age [12]. The prevalence of coalitions in the United States (US) has been reported from 1–13%; the wide range is due in part to the undetermined proportion of patients who remain asymptomatic [4]. It has been reported in the literature that as many as 75% of patients are asymptomatic [13]. Patients who are symptomatic typically endorse pain that is worsened with activity and improved with rest [4]. Increased stiffness and decreased range of motion may be concurrent with pain due to progressive ossification of the foot [4,14,15]. Furthermore, patients may also report flattening of the longitudinal arch with a valgus deformity of the hindfoot that accompanies or predates symptoms [6]. The location of pain in the foot usually depends on the specific tarsal coalition, with talocalcaneal coalitions presenting with migrating medial malleolus pain and calcaneonavicular coalitions causing pain over the anterior process of the calcaneus, in the talus, or more distally [12,16]. Patients may also present with a spastic peroneal flatfoot, but there is debate within the literature on the positive predictive value of this presentation in identifying a tarsal coalition and the frequency with which patients present with this abnormality [1,17].

In addition to pain and stiffness, tarsal coalitions can cause aberrancies in normal foot and ankle biomechanics or may be seen in conjunction with foot and ankle fracture patterns. While multiple case reports and incomplete literature reviews have been published discussing fracture in the presence of tarsal coalition, a comprehensive review with thorough discussion of related biomechanical factors and their contribution to fracture patterns is indicated. Thus, the purpose of this review is to discuss the biomechanics seen with tarsal coalitions and to describe reports of fracture occurring concomitantly with tarsal coalitions. We will discuss diagnostic options and treatment approaches in the setting of a fracture with a preexisting tarsal coalition.

## 2. Methodology

**Figure 3:**
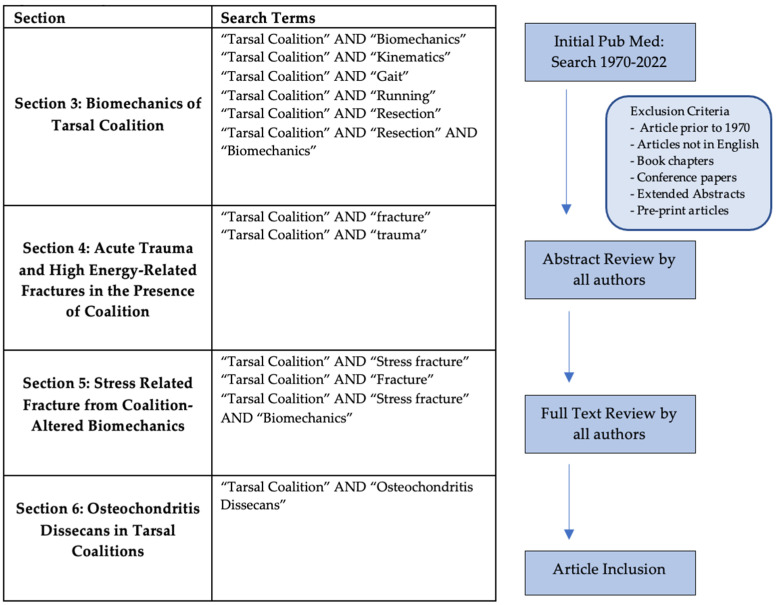
Study Selection and Search Criteria.

Articles in each individual section were found via a PubMed term search between 1970–2022 or via the references in articles from the PubMed search. Search terms and inclusion criteria for each section were utilized as described in Figure 3. Articles were excluded if they were written in a non-English language, and if they were a book chapter, conference paper, extended abstract, or pre-print. All abstracts were reviewed by authors followed by a full text review prior to inclusion.

## 3. Biomechanics of Tarsal Coalition

Tarsal coalitions have significant and clinically relevant effects on foot and ankle biomechanics and gait. With talocalcaneal and calcaneonavicular coalitions, the two most common types of tarsal coalitions, the rotary and gliding motion of the talus against the calcaneus is restricted [18]. When examined in the coronal plane, the gait of patients with a tarsal coalition demonstrates a significantly restricted subtalar range of motion and increased subtalar angular velocity [18]. Due to these changes in subtalar kinematics, patients have restrictions in eversion and inversion of the foot and a shortened time interval from heel strike to maximum eversion, both of which increase the magnitude of impact during locomotion [18]. The difference in plantar pressure specifically in patients with tarsal coalitions has also been examined. Prior to surgical resection, patients with tarsal coalitions were found to have significantly higher medial midfoot pressures during walking and running compared to normal controls [19]. The altered biomechanics found in tarsal coalitions have been hypothesized to contribute to stress on neighboring bony anatomy and an increased risk of fracture [20,21].

Although tarsal coalition resection can improve pain and increase return to activity rates, whether patient gait biomechanics improve following surgery is less clear. Significant improvements in passive range of motion have been found in patients after tarsal coalition resection, but range of motion typically remains lower than normal in patients without previous coalitions [17,22,23]. Prior work examining foot kinematics demonstrated that following tarsal coalition resection, patients continue to have significantly reduced subtalar range of motion when compared to normal feet with no significant difference between pre-operative and post-operative motion [18]. The continuation of these altered biomechanics indicates that patients with tarsal coalitions have similar eversion-inversion motion restrictions even after surgical intervention [18]. These differences in inversion and eversion mobility can have functional consequences. Chambers et al. found a positive correlation between side-to-side mobility of the foot and functional scores based on functional tests such as single-limb standing and gait analysis [23].

When examining the effect of surgical intervention on plantar pressure, the results are mixed. A study by Hetsroni et al. found that tarsal coalition resection reduces elevated medial midfoot pressures to those of normal feet during walking [19]. However, patients continued to have persistently elevated medial midfoot mean pressures and impulses during running following surgery, indicating that running accentuates pathological loading [19]. In contrast, a study by Lyon et al. found that following tarsal coalition resection, patients continued to have significantly greater peak pressures in the midfoot and first metatarsal head compared to normal feet during walking [24]. Furthermore, patterns of muscle activity in the lower extremities remain persistently aberrant after tarsal coalition resection. Electromyography studies have found that despite close to normal gastrocnemius, peroneal, and soleus muscle strength, patients after tarsal coalition resection demonstrate continued abnormal premature and prolonged firing in the peroneus longus during walking; this aberrant activity often extended to the gastrocnemius and soleus [24].

## 4. Acute Trauma and High Energy-Related Fractures in the Presence of Coalition

Altered biomechanics resulting from tarsal coalitions, particularly due to subtalar joint rigidity, are thought to increase stress across structures adjacent to tarsal coalitions [25,26]. Over the last few decades, several case reports have documented acute traumatic and high energy-related fractures in the presence of preexisting talocalcaneal and calcaneonavicular coalitions [27,28,29,30,31]. Typically, tarsal coalitions are incidentally found after identification of the fracture with conventional radiographs for calcaneonavicular coalitions [10]. Additional cross-sectional imaging may be required for identifying subtle talocalcaneal forms or differentiating osseous, fibrous, or cartilaginous coalitions with magnetic resonance imaging (MRI) [10].

Although there are no established clinical guidelines on the treatment of tarsal coalitions in the setting of acute traumatic fractures, the decision to concomitantly address a coalition in the reported cases to date has been influenced by the presence of pre-existing symptoms and patient characteristics (e.g., age, activity), characteristics of the coalition (e.g., calcaneonavicular versus talocalcaneal), detection of degenerative changes in which arthrodesis could be performed, and the need for coalition resection to obtain adequate fracture reduction [27]. Three cases of calcaneus fractures (two closed intraarticular and one open, comminuted) in the setting of middle facet coalition (two bony, one fibrous) have been reported in the literature [27,29,30]. All three patients were treated with ORIF, of which two did not undergo excision or arthrodesis of talocalcaneal coalition due to lack of pre-traumatic symptoms while one 50-year-old male patient with preexisting mild hindfoot pain and degenerative changes in the subtalar joint received concomitant subtalar arthrodesis.

Three case reports of fractures through the calcaneonavicular bar with non-operative management of the fracture and variable treatment of the concomitant calcaneonavicular coalition have been documented [27,29,30]. Pai et al. present the case of a 43-year-old female patient treated nonoperatively in a splint for six weeks with subsequent union and return to work at the four-month follow-up visit [28]. In contrast, despite the union of the calcaneonavicular bar fracture with conservative cast treatment, one 17-year-old male had persistent pain requiring triple arthrodesis [32]. Finally, Tanaka et al. demonstrated a successful return to truck driving for a 23-year-old male after en bloc resection from the beak of the calcaneus to the fracture line. Although the authors acknowledged that conservative treatment may have been adequate, they opted for resection due to the possibility of delayed union due to subtalar joint rigidity and refracture through the calcaneonavicular coalition [31].

Two case reports of three patients with fracture of the sustentaculum tali adjacent to talocalcaneal coalitions have been published, with excellent postoperative functional outcome scores after coalition resection in all three patients [25,33]. Kehoe and Scher present two pediatric patients (11 and 12 years old) with different treatment methods for sustentaculum tali fractures: one with concomitant coalition resection and excision of a fracture fragment after chronic nonunion, and one acute fracture with resection performed after six weeks of immobilization and non-weight-bearing to provide time for healing of the acute fracture [25]. These authors surmised that in the acute setting, a simultaneous excision of both the fracture fragment and tarsal coalition would have rendered the hindfoot unstable and likely to collapse into varus. In addition, simultaneous fracture fixation and tarsal coalition excision would have been technically challenging, given inadequate sustentaculum tali fracture components to repair [25].

Further case examples include the case of a 23-year-old patient with persistent inability to weight-bear after an ankle sprain [31]. The patient was found to have a fracture through a talocalcaneal coalition and was treated with an immobilizing orthosis and weight-bearing as tolerated [31]. Hughes and Brown present a 32-year-old male with a vertical fracture through the posterior third of the talar body with posteromedial displacement of roughly 7 mm in the setting of a talocalcaneal coalition. In this case, the patient’s osseous coalition had to be excised to achieve an adequate reduction of the fracture. At 1-year postoperatively, this patient returned to sports without osteonecrosis of the talus and only moderate hindfoot motion restriction [34].

Two cases of ankle fractures, one involving the tibial pilon and one involving both the medial and lateral malleoli, have been documented with incidental findings of talocalcaneal coalitions. Both patients underwent operative fixation of the fracture (one open and one percutaneous with arthroscopic guidance) and without excision of the coalition as neither patient had pre-traumatic symptoms [35,36]. One patient (53-year-old female) had subsequent hardware removal without increased pain or arthritic changes at 15 months postoperatively while another (16-year-old male) had no pain but was unable to invert his hindfoot.

## 5. Stress Related Fracture from Coalition-Altered Biomechanics

Stress fractures occur in the presence of repetitive mechanical stress on an affected bone, such as in the case of overuse [37]. They are a common pathology, accounting for up to 10% of all orthopedic injuries and up to 20% of injuries seen in sports medicine clinics [38]. The two imaging modalities commonly used to evaluate stress fractures in the foot and ankle are radiography and MRI, with the latter having a significantly higher sensitivity and specificity, especially in the early stages of injury [37,39]. Typical treatment for stress fractures consists of activity modification, analgesics, and potential bracing until pain symptoms resolve [40].

Although rare, stress fractures can also be seen in association with tarsal coalition [20,41,42,43,44]. It is thought that this associated stress response may be due to altered biomechanics of the foot leading to increased, abnormal load transfer and hindfoot stress-loading, leading to subsequent fracture [20]. In the case series reported by Jain et al., all six of the adolescent patients presented with diffuse pain of insidious onset and hindfoot stiffness [20]. All had a tarsal coalition of the fibrous sub-type. The locations of stress fractures or stress responses in the patients were the posterosuperior calcaneus, the posterior calcaneus, the cuboid and head of talus, the base of the third metatarsal, the posterosuperior calcaneus, and the head of talus. After using MRI to delineate stress fracture with coalition from coalition alone, the patients were started on nonoperative treatment including analgesics, activity modification and either orthotics or shoe modification [20]. Because tarsal coalitions are often found incidentally, a better understanding of the relationship between coalitions and stress response would aid in swift diagnosis and treatment.

Other case reports of talar stress fracture with preexisting talocalcaneal coalition exist in the literature. Manzotti et al. present a 24-year-old non-professional female runner presenting with left hindfoot pain without a specific area of point tenderness [41]. Initially, radiographs and CT scans were obtained and were non-illustrative. Cancellous edema of the talus due to stress fracture was revealed upon review of MRI, highlighting the utility of this imaging modality in diagnosis of stress injuries [41]. Similar to the six patient case series described above, this patient was started on nonoperative treatment, which included NSAIDs, non-weight bearing, compressive dressing, ankle training, and the use of a wooden-soled shoe [20]. With this conservative approach, the patient experienced a complete resolution of symptoms and a high degree of satisfaction with no further complications.

A case report of a concomitant calcaneal stress fracture with a rare subtalar facet coalition was documented by Moe et al. in 2006 [42]. In this article, a 48-year-old woman presented with worsening left heel pain without any prior accident or injury, a common manifestation of tarsal coalitions. Similar to the case study by Manzotti et al., initial radiographs were unreliable [41,42]. The early clinical diagnosis was presumed to be plantar fasciitis; when the patient did not respond to conservative treatment, further imaging including MRI revealed a “posterior subtalar facet coalition with associated medial and lateral calcaneal stress fractures.” MRI allowed for detection of the osseous prominence that gave a “humpback appearance” of the superior posterior calcaneus on the lateral radiograph that was previously missed. These authors hypothesized that the calcaneal stress fracture was due to abnormal forces placed on the hindfoot due to coalition [20]. The treatment plan indicated for the patient to be partially weight-bearing with crutches, but there were no mentions of other treatments, follow-ups, or further complications.

Two cases of stress fractures associated with calcaneonavicular coalitions have been reported. Nilsson & Coetzee investigated a 47-year-old man with a 5-week history of pain in lateral aspect of his left foot that was exacerbated by completing a marathon [43]. Initial radiographs of the foot did not show any fracture or dislocation, but an MRI three weeks after the initial radiograph showed a fracture of the anterior process of the calcaneus and a fibrous calcaneonavicular coalition. A comparison MRI five weeks later showed the continued presence of bone marrow edema confirming the stress fracture. Similar to the previous cases, this stress fracture was treated non-operatively, incorporating non-impact and low-impact training with pool and elliptical trainer workouts for 2 months. The patient was able to run without discomfort, even later completing a marathon without symptoms. Pearce et al. documented a case of a 30-year-old rugby player with a 5-week history of foot pain [44]. An MRI scan was conducted first, which demonstrated some degenerative changes between the navicular and calcaneus. However, a CT scan clearly revealed a stress fracture across the anterior process and a fibrous calcaneonavicular coalition [44]. In contrast to the other cases, these authors decided that the risk of non-union of the stress fracture or recurrence was too high to treat conservatively and opted for surgical excision of the coalition and fixation of the fracture. The patient did well and returned to rugby 6 months later.

## 6. Osteochondritis Dissecans in Tarsal Coalitions

In addition to abnormal talocrural stress, hindfoot malalignment, ankle sprains and fractures, another possible co-occurring pathology with tarsal coalition is osteochondritis dissecans (OCD) of the talar dome. Cheng et al. aimed to determine the prevalence of OCDs among patients with tarsal coalition [45]. After studying ankle MRIs in 57 patients with tarsal coalitions, the study found 89% of these tarsal coalitions to be non-osseous and talar OCDs present in 29 of them. The authors concluded that talar OCD prevalence is higher in patients with tarsal coalition than the general population, attributing this occurrence to the altered biomechanics and repetitive talocrural stress due to the altered subtalar motion [45]. There is a paucity of research evaluating for presence of OCD in conjunction with tarsal coalition, and future research is indicated to further expand upon this phenomenon.

## 7. Discussion and Treatment Considerations

Foot and ankle injury in the form of fracture or sprain in the presence of an existing tarsal coalition is a relatively uncommon clinical entity. However, a large number of case reports outline this condition, with heterogeneous treatment options described throughout the literature. Our goal in this review was to provide a comprehensive summary of what has been published to date with regard to the co-occurrence of fracture and tarsal coalition. Given the wide array of management strategies employed to treat this condition, defining specific clinical algorithms for workup and intervention remains elusive. Moreover, given that the existing literature is limited to single patient case reports or small series, there is a high likelihood for an element of publication bias, with only positive outcomes being reported. Despite this, through careful review of the literature, some overarching principles can be gleaned for the treatment of this condition.

For patients being evaluated for foot and ankle fracture in the presence of an incidentally noted or previously symptomatic tarsal coalition, the first branch point for management should be to determine whether the fracture is acute, resulting from a recent injury, or chronic and stress-related in nature. If the fracture is stress-related and appears to be related either to altered biomechanics resulting from the tarsal coalition or from a period of intense activity increase leading to subsequent overuse, nonoperative management should trialed. Nonoperative treatment modalities which have been reported with success in the literature include NSAIDs, non-weight-bearing, compressive dressing, ankle training, and the use of a wooden-soled shoe [20]. Casting and activity modification as well as partial weight-bearing with crutches have also been utilized with positive results [42]. Except in cases of prolonged duration of pain after a trial of nonoperative management and persistent CT-confirmed nonunion, we recommend avoidance of surgical intervention for stress related fracture in the setting of tarsal coalition.

In patients who present after an acute trauma, with a fracture either to the foot or ankle, who are also found to have a tarsal coalition to the ipsilateral extremity, we recommend careful consideration of patient and fracture specific factors to guide treatment. While nonoperative management is not commonly appropriate for acute fracture in the setting of tarsal coalition, fractures which are minimally displaced, where an incidentally noted, asymptomatic tarsal coalition is present, may be managed without surgery. In this case, we recommend a period of casting and non-weight-bearing to prevent fracture displacement. Fractures involving the calcaneus, talus, tibial plafond, medial and lateral malleoli, and bones of the midfoot which are significantly displaced should be treated operatively, following standard operative protocols for these respective injuries. Any existing coalition should be evaluated through the use of advanced imaging modalities. In cases where a coalition is diagnosed and a preoperative examination is possible, a careful history should be obtained as to whether the tarsal coalition is currently symptomatic or has caused a period of symptoms at an earlier time point. Physical exam, including point tenderness at the site of the coalition or range of motion limitation from the fibrous or bony union, may be significantly limited given swelling and pain in the acute traumatic setting.

If the tarsal coalition is confirmed to be currently symptomatic or to have caused a period of prior symptomology, the coalition should be resected at the time of fracture fixation with either subcutaneous fat, extensor digitorum brevis, or bone wax interposition to prevent coalition return. However, as Kehoe et al. caution [25], tarsal coalition excision should only be performed if there is no concern for further destabilization of the midfoot or hindfoot, as achieving an appropriate reduction with adequate stability is paramount to success of fracture management, and takes precedence over coalition resection. A final consideration should be given for the presence of pre-existing osteoarthritis at the subtalar, calcaneocuboid, or talonavicular joint, especially in older patients with pre-existing pain with inversion and eversion of the ankle. In patients with symptomatic arthritis, joint arthrodesis procedures can be combined with ORIF to achieve a stable, plantigrade foot and to reduce postoperative pain and need for reoperations at the expense of joint range of motion.

## 8. Conclusions

Tarsal coalitions are an uncommon foot pathology that may be asymptomatic or present with vague pain and progressive stiffness. Abnormal loading forces of the mid- and hindfoot prior to surgery may contribute to recurrent ankle sprains or fractures. These altered biomechanics may still be present following surgical intervention with activities that increase the load placed on the foot and ankle, such as in running or jumping. Multiple case series have described the association of these coalitions with stress fractures, with key findings including the utility of nonoperative treatment and the superiority of MRI in visualizing these stress responses. Osteochondritis dissecans is another pathology that may commonly be seen in conjunction with tarsal coalitions, given abnormal joint biomechanics and high incidence of ankle sprains. Why some coalitions remain asymptomatic while other lead to progressive pain, stiffness, and fractures is unclear, but is a potential avenue for further research. This study summarizes the available literature regarding fracture in the presence of concomitant tarsal coalition and discusses possible nonoperative and surgical treatment to improve pain, function, and quality of life for patients with this rare, but important association.

## Data Availability

Not applicable.
